# The Guidelines International Network Public Toolkit: Supporting Guideline Developers to Implement Best‐Practice for Patient and Public Involvement

**DOI:** 10.1002/gin2.70084

**Published:** 2026-08-26

**Authors:** Sarah Scott, Anneliese Synnot, Karen Graham, Lyubov Lytvyn, Dawid Pieper, Mark Rasburn, The GIN Public Working Group

**Affiliations:** ^1^ National Institute for Health and Care Excellence (NICE) Manchester UK; ^2^ School of Public Health and Preventive Medicine Monash University Melbourne Australia; ^3^ Department of Public Health Centre for Health Communication and Participation La Trobe University Melbourne Australia; ^4^ Scottish Intercollegiate Guidelines Network (SIGN) Edinburgh UK; ^5^ Department of Health Research Methods, Evidence, and Impact McMaster University Hamilton Ontario Canada; ^6^ Brandenburg Medical School (Theodor Fontane), Institute for Health Services and Health System Research, Faculty of Health Sciences Brandenburg Rüdersdorf Germany

**Keywords:** GIN Public Toolkit, guideline development, guideline quality, patient and public involvement, stakeholder engagement

## Abstract

Patient and public involvement (PPI) is increasingly recognised as essential to the development of trustworthy and relevant clinical guidelines. However, guideline developers face persistent challenges in implementing best‐practice PPI, including developing an appropriate PPI strategy, recruiting and supporting patients, and including diverse perspectives. The Guidelines International Network (GIN) Public Working Group developed the GIN Public Toolkit to address these challenges and support meaningful involvement across the guideline development lifecycle. This commentary outlines the Toolkit's background, purpose, structure, global impact and future directions. Freely available online, the Toolkit provides practical guidance and case examples based on the experiences of guideline developers around the world. Since its launch in 2015, the Toolkit has evolved to include 10 chapters delivered via dual‐format: e‐print and a website. Website analytics and citation data indicate the Toolkit's growing global reach, supported by the ongoing chapter translations, training and dissemination activities. The Toolkit's vision is to remain a living resource that is shaped by collaboration and is responsive to guideline developer's needs. Future directions include increasing the Toolkit's accessibility through further translations, refining citation practices and targeted promotion and dissemination strategies. The GIN Public Toolkit continues to build global capacity for meaningful PPI in guideline development.

## Summary

- Supports best‐practice patient and public involvement: The GIN Public Toolkit provides practical, evidence‐informed guidance to help guideline developers embed meaningful patient and public involvement across the guideline lifecycle.
- Addresses real‐world challenges: The Toolkit tackles common challenges with patient and public involvement, including recruitment, support, communication and diversity, with actionable solutions and case examples.
- Evolving and user‐driven: Regularly updated in response to user feedback, the Toolkit reflects emerging practices such as living guidelines and virtual involvement.
- Global reach and impact: With translations, international uptake and use in training, research and policy, the Toolkit is a key resource for building global capacity in patient and public involvement.

## 1 The History and Importance of PPI in Healthcare Guideline Development

In the late 1900s, clinical guidelines were largely developed by clinicians and researchers without patient input. This clinician‐centric approach was criticised for excluding diverse patient values and preferences that could lead to healthcare recommendations that were biased, misaligned with patient needs and potentially harmful [1]. In 2003, AGREE I, a guideline quality assessment tool, was developed and included stakeholder involvement as a standard [2], although the involvement of patients in guideline development was still inconsistent in practice [3]. A turning point was recognised in 2011 when the Institute of Medicine published a set of standards for guideline development, that outlined the integration of patient and public involvement (PPI) within different stages of guideline development, including as a member of the guideline development group [4]. 'Patients' are individuals with personal experience of a condition or service, including users, carers or their representatives, while the 'public' refers to members of the wider community who may be affected by health and care services indirectly or as citizens and taxpayers.

Since then, PPI in guideline development has gradually become accepted and incorporated into guideline development [5]. A published needs assessment of guideline developers around the world indicated that 81.5% of 111 developers perceived the incorporation of the patient voice as a relevant need for their organisation [6]. PPI is perceived as necessary to bridge scientific knowledge and lived experience of patients and carers. PPI aims to improve the relevance and quality of clinical guidelines by ensuring that recommendations align with patient or population‐level needs, and thereby improving healthcare quality [7]. Furthermore, research indicates that PPI helps to increase the acceptability and trustworthiness, creates a sense of shared ownership and improves the adoption of guideline processes and recommendations [8, 9].

The evolution of PPI reflects a broader shift towards a more collaborative and person‐centred approach to guideline development. International bodies such as WHO, NICE, SIGN and NHMRC have developed fundamental frameworks and practical tools to embed meaningful involvement, equity and transparency in clinical guidelines [10, 11, 12, 13]. Involvement strategies often include recruiting one or more patient and public members to the guideline development group, but increasingly other methods such as advisory groups, workshops and surveys to capture diverse perspectives are being used [14, 15]. While GIN Public uses PPI as the preferred term, we acknowledge that there are related terms such as patient engagement, for example.

## 2 The Challenges of Implementing Patient and Public Involvement in Guideline Development

Despite the wide‐spread acceptance and value of PPI, many guideline organisations face persistent challenges to implementing best‐practice. Many organisations still exclude patients and the public from decision‐making groups [4], and those committed to inclusion often lack standardised procedural guidance on how to include patients and the public [15]. Even for organisations committed to PPI, there are several challenges to inclusion or harnessing meaningful involvement including:

- *systemic and resourcing constraints* for recruitment, training and support (e.g., insufficient funding, time or human resources);
- *recruitment issues*, including difficulty in recruiting patients and the public that meet eligibility and conflict of interest requirements;
- *power and communication imbalances* (e.g., lack of support from guideline developer organisations to increase capacity for health literacy; reduce jargon and increase plain‐language and improve accessibility requirements, particularly when involving certain groups in society such as children and young people or people with cognitive impairments);
- *role confusion and insufficient training* to ensure meaningful and legitimate involvement (e.g., patient and public members often report feeling uncertain about their role and impact without ongoing support);
- *diversity and representation issues*, including the challenge of harnessing diverse perspectives and representation of specific underrepresented patient populations due to cultural, socioeconomic, or health‐related barriers [16, 17].

This commentary introduces the GIN Public Toolkit—developed by the Guidelines International Network (GIN) Public Working Group—as a practical resource of guidance and case examples to support meaningful PPI in guideline development and implementation globally.

## 3 The GIN Public Toolkit

GIN founded the GIN Public Working Group in 2007 to advance effective PPI in guideline development globally. It is a working group of researchers, health professionals and patient and public members who promote ways to inform and involve the public in guideline activity around the world. Its creation was a response to the need for practical resources and standardised methods to support meaningful involvement of patients and the public in guideline development. A key output of this group is the GIN Public Toolkit [18], a freely available, evidence‐informed resource designed to support guideline developers in embedding standard methods and overcoming barriers to best‐practice in PPI across the guideline lifecycle.

The rationale for developing a toolkit specific to guidelines was that the methods, processes and tasks associated with each stage of a guideline are different and varied compared to the stages of a research project. The GIN Public toolkit attempts to outline these nuances that guideline developers need to consider when involving people within those specific contexts and guideline development tasks.

### 3.1 Toolkit Objectives

The main objectives of the toolkit are to:

1. Describe different methods for PPI in different stages of guideline development; including the advantages and disadvantages of these methods.
2. Provide best‐practice examples of PPI methods from research and experience from international guideline developers.
3. Describe the barriers, solutions and resources needed to support effective PPI in guideline development and implementation.

The toolkit is written by members of GIN Public. Patient and public representatives are included as co‐authors or as peer reviewers on individual chapters. Each chapter reviews any existing research or frameworks and includes case studies drawn from the practical experience of the author team.

### 3.2 Toolkit Evolution

First published in 2012, the Toolkit initially comprised of six chapters, with subsequent updates in 2015, 2021 and 2025 leading to the inclusion of 10 chapters to reflect Toolkit user feedback and evolving practice in PPI. Readers can find chapter summaries, key messages and top tips for each chapter online [18]. User feedback highlighted that an overview of the key challenges that each chapter addresses would be beneficial. Table 1 provides this along with an overview of the current chapters.

**Table 1:** GIN Public Toolkit chapter summaries and key challenges and issues addressed by each chapter.

Chapter title	Summary	Challenges and issues addressed
Consultation	Explores methods for gathering input from patients and the public, such as surveys, interviews and focus groups, to inform guideline development.	− Designing inclusive consultation processes − Identifying who to consult (public vs. targeted) − Choosing appropriate consultation methods (e.g., surveys, focus groups) − Managing and analysing consultation feedback − Integrating consultation findings into guideline development − Evaluating the effectiveness of consultation activities
Research	Discusses how to incorporate research on patient and public views, including primary studies and systematic reviews, into guidelines.	− Planning for the use of research on patient views − Identifying relevant types of research (qualitative, mixed methods) − Searching for and accessing patient‐centred evidence − Assessing the quality and certainty of evidence on patient views − Summarising and presenting research findings for guideline panels − Addressing gaps in available research − Avoiding tokenism in evidence use
Recruitment and Support	Offers strategies for recruiting patients and public contributors and supporting them effectively throughout the guideline development process. Addresses the known key barriers to involvement.	− Recruiting and involving diverse and representative patient/public contributors − Clarifying roles and expectations for contributors − Providing training and ongoing support − Addressing barriers to participation (e.g., time, confidence, accessibility) − Supporting virtual participation − Managing power dynamics within guideline groups
Role of the Chair	Describes the responsibilities of the guideline committee chair in fostering inclusive participation, managing dynamics and supporting patient and public involvement.	− Creating an inclusive and respectful group culture − Supporting patient/public members to contribute meaningfully − Managing group dynamics and balancing perspectives − Addressing challenges in communication and participation − Providing leadership that values lived experience
Systematic Reviews	Covers how to involve patients and the public in the planning, conduct and interpretation of systematic reviews used in guideline development.	− Involving patients/public in planning and conducting reviews − Identifying appropriate stages for involvement − Communicating complex review methods and findings − Ensuring meaningful contributions to evidence interpretation − Reporting and evaluating PPI in systematic reviews
Patient Information	Provides guidance on translating guideline recommendations into accessible, plain‐language information for patients and the public.	− Translating technical guideline content into plain language − Ensuring accessibility and health literacy − Co‐producing patient‐directed materials − Selecting relevant recommendations for patient audiences − Communicating risks, benefits and strength of recommendations − Designing user‐friendly formats − Evaluating the impact of patient information tools
Shared Decision Making	Explains how guidelines can support shared decision‐making between patients and healthcare professionals, including tools and implementation strategies.	− Embedding shared decision‐making (SDM) principles in guidelines − Identifying when SDM is most appropriate (e.g., preference‐sensitive decisions) − Presenting options and trade‐offs clearly − Supporting clinicians and patients in using guidelines collaboratively − Developing tools to facilitate SDM
Using Guidelines	Focuses on how patients and the public can use guidelines in real‐world settings, including barriers and facilitators to uptake.	− Promoting awareness and accessibility of guidelines − Supporting implementation in clinical and community settings − Addressing barriers to guideline uptake − Engaging patients/public in dissemination and adaptation − Evaluating the use and impact of guidelines
Patient Involvement in HTA	Discusses the role of patients and the public in health technology assessment (HTA), including methods for meaningful engagement.	− Clarifying the role of patients in health technology assessment (HTA) − Differentiating between patient input and patient‐based evidence − Addressing barriers to involvement in HTA processes − Applying Health Technology Assessment International values and quality standards − Ensuring early and continuous engagement − Demonstrating the impact of patient involvement
Patient and Public Involvement in Living Guidelines	Addresses how to maintain ongoing patient and public involvement in the development and updating of living guidelines.	− Sustaining engagement over time in continuously updated guidelines − Adapting involvement models to living guideline processes − Managing contributor fatigue and turnover − Supporting contributors with training and feedback − Prioritising updates based on patient/public input − Evaluating and improving ongoing involvement

## 4 International Impact

Since its inception, the GIN Public Toolkit has had a growing international impact by (a) extending its global reach through the development of digital editions and translations; (b) supporting capacity building in PPI methods for guideline developers; and (c) informing further research, training and organisational strategy for PPI in guideline development.

### 4.1 Extending International Reach

In 2021, the Toolkit was digitally adapted into an interactive website, in addition to a downloadable PDF [18]. We did this as we thought it would help readers navigate the growing content, allow material to be read across different devices, enable updates or new content to be added more easily and to gather more granular download data To enhance international accessibility, selected Toolkit chapters have been translated into 4 different languages: French (4 chapters), German (2 chapters), Japanese (full toolkit) and Chinese (1 chapter). Further translations are ongoing.

For January to October 2025, website analytics indicate that the toolkit received a total of 2409 visits (averaging 241 visits per month). In September and October 2025 toolkit users came from 75 countries (Figure 1). Users were from Africa (14 countries), Americas—North/Central (7 countries), Americas—South (6 countries), Asia/Middle East (22 countries), Europe (24 countries) and Oceania (2 countries). The wide geographical spread suggests that the Toolkit is becoming a recognised and trustworthy manual for PPI globally.

**Figure 1:**
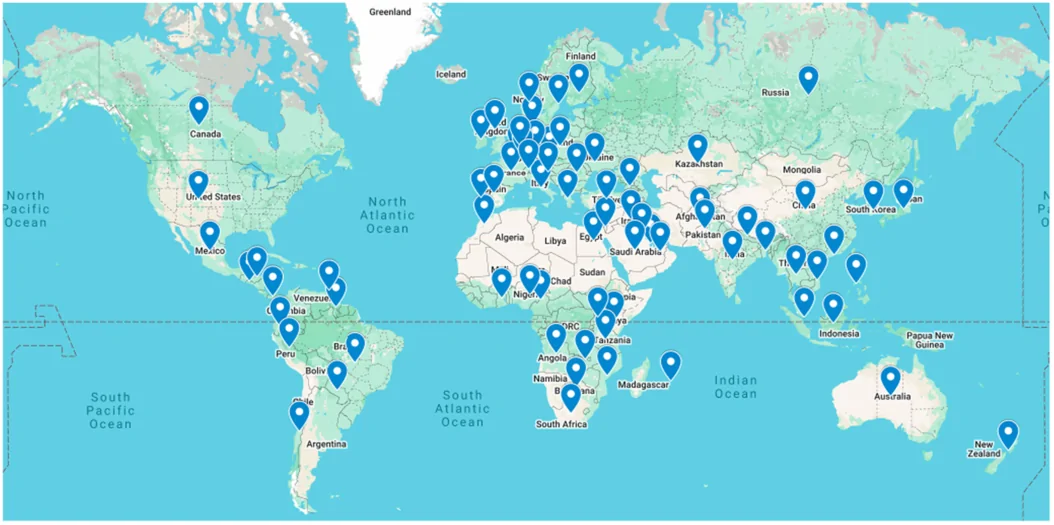
Geographical spread of Toolkit users in September and October 2025.

### 4.2 Capacity Building Through Training and Knowledge Dissemination

The GIN Public Working Group has delivered 12 international conference presentations or workshops at either the annual GIN conference, the Global Evidence Summit, or for GIN members since 2021. These sessions have supported capacity building among guideline developers, patient organisations and patient contributors. Topics have included:

- best‐practice methods for PPI;
- methods for shared‐decision making;
- developing patient information or patient decision‐aids;
- including research on patient and public views in guidelines;
- recruiting, supporting and overcoming barriers;
- real‐world experience as evidence;
- methods and strategies for involving patients and the public in living guidelines.

### 4.3 Informing Further Research, Organisational Strategy and Training Materials

A Google search for websites and articles using the term ‘GIN Public Toolkit’ in 2‐year periods since 2012 demonstrated an increase in the use of the Toolkit over time (Figure 2). The jump in citations from 2021 could indicate the success of the transition to digital format, increased accessibility of the toolkit and increased publicity through conferences and workshops.

**Figure 2:**
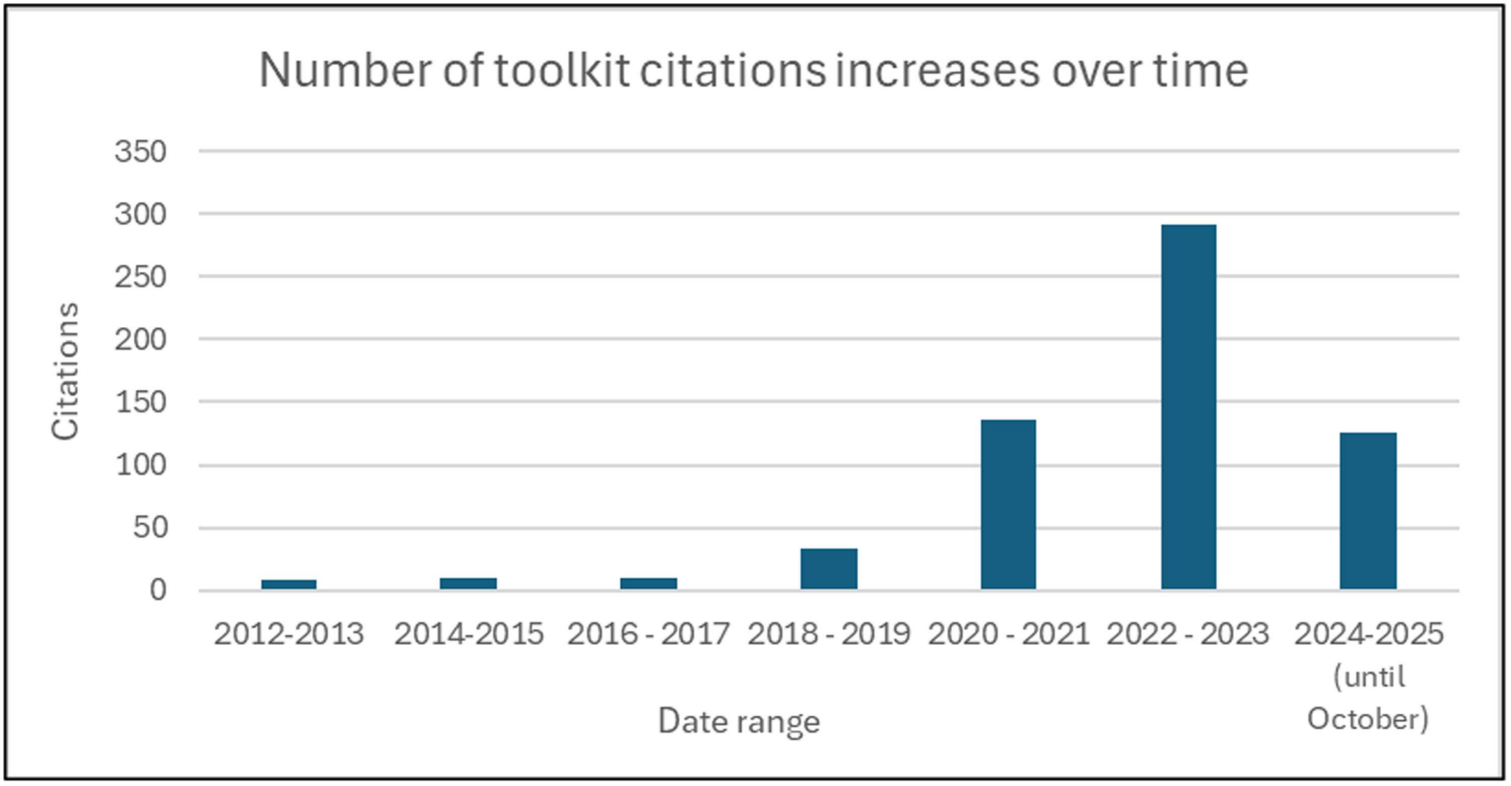
Number of toolkit citations.

We also investigated key uses of the Toolkit by performing a citation analysis in Google Scholar. First, we screened the first 5 pages of results (10 results per page) for each 2‐year period (e.g., January 2018 until December 2019). Among 60 citations (since 2024) the majority were about informing research and research protocols about the involvement of patients and the public in guideline development (e.g., Pfisterer‐Heise et al. [19]). Additionally, the GIN toolkit was found:

- as a referenced source within journals and conference presentations about how to involve patients and the public in guideline development (e.g., Loyola et al. [20]);
- to support training and educational materials around PPI in guideline development (e.g., Schaefer [21]);
- to inform the development of guideline quality checklists (e.g., Petkovic et al. [22], National Institute for Health and Care Excellence [23]); and
- to inform the PPI strategy within developer organisations (e.g., National Institute for Health and Care Excellence [23]).

## 5 The Vision and Goals of the GIN Public Toolkit

The GIN Public Toolkit is a living and evolving resource, designed to respond to the needs of guideline developers and considering any cultural or societal contexts. For example, in response to the shift from conducting in‐person to virtual guideline development meetings during the COVID‐19 pandemic, the ‘Recruitment and Support’ chapter was updated to reflect considerations for virtual involvement—an enhancement made at the request of guidelines developers.

GIN Public actively encourages guideline developers to contribute to the Toolkit's growth by requesting and developing additional content, such as new chapters, extensions of existing chapters and inclusion of best‐practice case studies within existing chapters. Feedback and suggestions are captured at activities like GIN workshops and webinars, via occasional surveys of GIN members and can also be made via an online survey on the website. These are reviewed semi‐regularly by the GIN Public Steering Group, which considers the evidence and rationale for change, impact on users, feasibility and resourcing. This collaborative approach ensures that the Toolkit is relevant to guideline developers and reflects evolving changes or innovation in PPI. The overarching goal is for the Toolkit to serve as a practical resource—used flexibly by developers to shape their PPI strategies and to address specific implementation challenges. As the Toolkit continues to evolve, its utility lies in its adaptability and its capacity to support guideline developers in embedding meaningful public involvement throughout the guideline development process.

## 6 Future Directions for the GIN Public Toolkit

GIN Public remains committed to ensuring the Toolkit is accessible, relevant and globally inclusive. This includes ongoing efforts to translate the Toolkit into additional languages and co‐develop content with international partners to reflect diverse PPI methods and processes. New chapters are being developed to focus on legal aspects of PPI (such as data protection and confidentiality) and defining and measuring PPI impact, in response to current gaps in practice and knowledge within the field.

To support wider adoption of the Toolkit, GIN Public is exploring promotion and marketing strategies, and further training and international workshops. A key priority is to document and share use‐cases and impact through case studies that clearly demonstrate how the Toolkit has been used.

As the Toolkit continues to grow, we anticipate that we will maintain the two formats (a digital e‐book version and web‐based version). However, this dual‐format approach presents challenges in terms of resourcing, version control and visibility. A clear marketing and communication strategy is needed to ensure guideline developers are aware of future updates. Current citable versions of the toolkit chapters (with date of publication) can be found in the resources section of the GIN website [24].

Furthermore, the living nature of the Toolkit, while a strength, also complicates consistent referencing. Recent publications have cited outdated versions of the Toolkit, despite significant updates being available online. This highlights the need for a clearer referencing system that enables users to cite specific online chapters and versions accurately. GIN Public aims to address these challenges by developing a robust citation framework, enhancing discoverability and continuing to engage with the global guideline development community to ensure the Toolkit remains a trusted and practical resource.

## 7 Conclusion

The GIN Public Toolkit has become an important resource for guideline developers seeking to embed meaningful PPI across the guideline lifecycle. With a focus on relevant practical and evolving content or tools (shaped by user feedback and co‐production), the Toolkit has supported capacity building for implementing or researching meaningful PPI methods and processes on an international scale.

The Toolkit's vision is to remain a living resource, shaped by collaboration and responsive to guideline developer's needs. Future directions include increasing the Toolkit's accessibility through further translations, refining citation practices and targeted promotion or dissemination strategies. The GIN Public Toolkit continues to build global capacity for meaningful PPI in guideline development.

## Author Contributions

**Sarah Scott:** conceptualisation, methodology, formal analysis, project administration, supervision, writing – original draft, writing – review and editing, investigation. **Anneliese Synnot:** conceptualisation, writing – original draft, writing – review and editing. **Karen Graham:** writing – review and editing, conceptualisation. **Lyubov Lytvyn:** writing – review and editing. **Dawid Pieper:** investigation, writing – review and editing, formal analysis. **Mark Rasburn:** writing – review and editing.

## Funding

The authors have nothing to report.

## Ethics Statement

The authors have nothing to report.

## Conflicts of Interest

S.S., A.S., K.G., L.L. and M.R. have co‐authored one or more chapters of the GIN Public Toolkit. All authors are members of the GIN Public Working Group. A.S. is a member of the Editorial Board of Clinical and Public Health guidelines but did not participate in the review or the editorial process of this article and had no influence on the Editorial decisions related to it.

## Data Availability

Data sharing is not applicable to this article as no data sets were generated or analysed during the current study.
